# Reliability and Validation of the Japanese Version of the Patient Empowerment Scale

**DOI:** 10.3390/healthcare10061151

**Published:** 2022-06-20

**Authors:** Yoshihito Tsubouchi, Akiyoshi Tainosho, Koudai Shimomura, Kyosuke Yorozuya, Motoasa Kou, Rie Tsubouchi, Hiroyuki Tanaka, Yasuo Naito

**Affiliations:** 1 Department of Rehabilitation, Faculty of Health Science, Naragakuen University, Nara-shi 631-8524, Japan; tsubouchi@naragakuen-u.jp; 2 Department of Rehabilitation, Akitsukounoike Hospital, Gose-shi 639-2273, Japan; akiyoshi.tainosho@gmail.com (A.T.); koudai0727@gmail.com (K.S.); 3 Faculty of Care and Rehabilitation, Seijoh University, Tokai-shi 476-8588, Japan; yorozuya.kyousuke@indigo.plala.or.jp; 4 Department of Psychiatry, Akitsukounoike Hospital, Gose-shi 639-2273, Japan; koumotoasa2004@gmail.com; 5 Department of Nursing, Akitsukounoike Hospital, Gose-shi 639-2273, Japan; rietsubouchi1014@gmail.com; 6 Graduate School of Comprehensive Rehabilitation, Osaka Prefecture University, Habikino-shi 583-8555, Japan; hytanaka@rehab.osakafu-u.ac.jp

**Keywords:** older patients, geriatric health, patient empowerment scale, Japan

## Abstract

Empowerment scales for inpatients have been developed worldwide, but their validity and reliability have not been adequately tested and applied to the health promotion and care among older adults during hospitalization. In this study, the Patient Empowerment Scale developed by Faulkner was translated into Japanese, and Japanese patients were surveyed to test its clinical applicability. To test its applicability, 151 patients in rehabilitation wards were surveyed in four municipalities. After considering ceiling/floor effects and validating the structure, the Patient Empowerment Scale—Japanese comprised 37 items and six factors: subject–staff interaction, environmental adjustment through collaboration, necessary information gathering and problem awareness, proactive behavioral practices, self-disclosure, and self-management of activities. Criteria-related validity assessment confirmed the scale’s correlation with the Health Locus of Control Scale, General Self-Efficacy Scale, 13-item Sense of Coherence Scale, Rosenberg Self-Esteem Scale, and Philadelphia Geriatric Center Morale Scale. Regarding internal consistency, the Cronbach’s alpha was 0.93 for all 37 items. The Cronbach’s alphas for the six factors were 0.93, 0.91, 0.92, 0.92, 0.91, and 0.75, respectively. In our test/re-test of reliability, Spearman’s rank correlation coefficient between the first and second total scores was ρ = 0.96, *p* < 0.01. These results confirm the scale’s validity and reliability, and its applicability to older hospitalized patients.

## 1. Introduction

In East and Southeast Asia, including Japan, the number of people aged ≥65 years is increasing rapidly and is expected to grow from 11% of the population in 2019 to 24% in 2050. This situation highlights the need to focus on efforts that maintain and promote the health and welfare of older adults as an urgent issue [1].

Recent research has shown that the onset of diseases, such as chronic obstructive pulmonary disease and dementia, can cause changes in older adults’ relationships and their sense of self-identity, both of which can lead to a feeling of powerlessness [1,2]. Furthermore, studies have demonstrated that older adults who have been hospitalized or institutionalized after the onset of an illness gradually begin to exhibit “learned helplessness and inactivity.” This means that they come to rely on physicians and medical personnel to make decisions about their treatments and life decisions [1,2,3]. To prevent this, we need to raise awareness among this population concerning their potential to adjust their behavior to maintain and improve their health, and to retain the ability to choose various medical and health welfare services (namely to promote personal empowerment) [4].

The concept of empowerment has been applied in various fields and populations, such as public health, welfare, and mental health, and has been defined in various ways in each field in terms of disease characteristics [5,6]. In the medical field, the empowerment concepts of Funnell and Anderson [7], Anderson and Funnell [8], Lau [9], and Aujoulat, D’Hoore, and Deccache [10] are often used in relation to patients with diabetes, as well as for those with other chronic diseases [11]. For older adults, Gibson’s concept of empowerment as “a social process of recognizing, promoting and enhancing peoples’ abilities to meet their own needs, solve their own problems and mobilize the necessary resources in order to feel in control of their lives” [12] (p. 359) has been widely applied [11]. However, scholars have pointed out that it is difficult to develop a scale to measure empowerment due to the diversity within the concept. This limitation has become an impediment to the widespread usage of the empowerment concept [13].

In recent years, culture- and individual-specific scales have been developed, such as the Diabetes Empowerment Scale (DES) and Empowerment Scale (ES) for patients with mental illness [14,15,16]. In addition, the Patient Empowerment Scale (PES) was developed to encompass older patients, where age, the amount of assistance needed with daily activities, and quality of daily care were identified as influential factors in the empowerment of older patients [17]. However, empowerment scales for older patients, including the PES, have been used despite the lack of sufficient confirmation of their reliability and validity. Furthermore, it has been noted that the application and interpretation of results differ among countries and cultures. Therefore, there is a need to establish the validity and reliability of the scales and evaluate their cross-cultural validity [15,18].

## 2. Background

In Japan, where the birthrate is the lowest in the world and the population is aging, the decline in healthy life expectancy due to confinement and weakness resulting from hospitalization has become a social problem. However, as yet, there is no evaluation scale that measures the empowerment of hospitalized older patients in Japan and, consequently, no support has been developed for such patients either. Although an empowerment scale for older adults, that is, for those living in community dwellings in Japan, has been developed [4,19], the questions on this scale were designed for healthy older adults living at home. Therefore, factors affecting the empowerment of hospitalized older patients in Japan, as well as specific support methods and their effects, have not been assessed.

The percentage of the world’s older population is expected to grow; therefore, support for collaboration and environmental adjustments between older patients and healthcare professionals is needed, based on shared self-determination and with the aim of ensuring independent living. The PES, which has been considered highly useful, is seen as a suitable patient-centered measure to evaluate the human and physical care environment and promote the empowerment of older patients. However, except for internal consistency and content validity, the PES has not been comprehensively tested, and evaluation of its validity and reliability was insufficient [15,18]. As a result, the use of the PES and the interpretation of its results differ among countries and regions, making continuous validation and comparison with results from other countries difficult. To address the situation in Japan, this study translated the PES, as an empowerment scale for hospitalized older patients, into Japanese. The Japanese version of the PES was then used to conduct a detailed evaluation of the validity and reliability of the scale, which had not been done in the original version, to enable its clinical application to older patients.

## 3. Materials and Methods

### 3.1. Design

The PES developed by Faulkner [17] was translated into Japanese (PES-J) using International Society for Pharmacoeconomics and Outcomes Research (ISPOR) task force procedures [20]. The quality of the translated PES-J scale was verified according to the Consensus-based Standards for the Selection of Health Measurement Instruments (COSMIN) checklist [21,22]. The PES-J uses a four-point Likert-type scale to measure each item (ranging from 0 = never to 3 = always), with 20 empowerment items initially added and 20 disempowerment items initially subtracted in relation to the total score, following Faulkner’s scoring method. The total score ranges from −60 to 60. We first examined ceiling and floor effects to assess bias in responses to the questionnaire items. For items that showed ceiling or floor effects, we qualitatively evaluated their effects on content and the scale’s structure.

Next, we tested structural and criteria-related validity [23]. For structural validity, exploratory factor analysis was conducted. In addition, confirmatory factor analysis was performed on the resulting model and compared to the original version of Faulkner’s two-factor model. In terms of criteria-related validation, no empowerment scale (gold standard) has yet been developed for older hospitalized patients in Japan. Therefore, we examined the relationship between the total PES-J score and scores of the factors using five scales that measure locus of control, self-efficacy, sense of coherence, self-esteem, and subjective well-being, which have been confirmed to be related to the concept of empowerment in previous studies [11,24,25].

For locus of control, we used the Health Locus of Control Scale (HLC), referring to the multidimensional health locus of control concept developed by Wallston, Strudler Wallston, and DeVellis [26] and taking into account Japanese culture, religious beliefs, and traditions [27]. The HLC comprises two factors (internal and external) and 14 items, with a full score of 56 points. The higher the total score, the stronger the influence of the internal items. For self-efficacy, we applied the General Self-Efficacy Scale (GSE) developed by Sherer et al. [28], which has been translated into Japanese [29]. The GSE comprises one factor and 23 items. A full score totals 115 points, and a high score indicates a high level of self-efficacy. For sense of coherence, we applied the 13-item Sense of Coherence Scale (SOC-13), developed by Antonovsky [30] and validated in Japanese [31]. The scale comprises 13 items with three factors—graspability, processability, and meaningfulness—and a full score of 91 points; the higher the score, the better the ability to cope with stress [30,31]. The Rosenberg [32] Self-Esteem Scale (RSES) was used to assess self-esteem [33]. The RSES comprises one factor and 10 items, with a perfect score equalling 50 points, and a high score indicating high self-esteem [32,33]. Finally, for subjective well-being, we adopted the Philadelphia Geriatric Center Morale Scale (PGC) as developed by Lawton [34]. The PGC comprises three factors and 17 items, such as psychological agitation, attitude toward aging, and loneliness and dissatisfaction, with a perfect score equalling 17 points, and a high score indicating high subjective well-being [35]. We examined internal consistency, test–retest reliability, and measurement errors in the reliability validation.

### 3.2. Method

The survey was conducted among older patients who presented with disease and who had experienced changes in their condition and daily activities over a short period of time to assess whether the PES-J could address environmental changes. Many health-related patient-reported outcome measures apply an interval of two weeks to three months. In this study, two weeks were considered a reasonable period [36,37]. Thus, for test–retest reliability, a second survey was conducted and analyzed two weeks after the first survey (one week after admission).

The patients were in convalescent rehabilitation wards that aimed to enable their functioning in independent community life within 60 days after the onset of various diseases, such as central nervous system, orthopedic, and internal diseases. These patients require active rehabilitation for a maximum of three hours per day and a maximum of 180 days after the onset of illness [38]. Concerning the sample size when recruiting participants, we adopted a target of ≥100 cases based on the COSMIN checklist.

We surveyed patients aged ≥65 years who had been admitted to convalescent rehabilitation wards located in four municipalities in Japan (Gose City, Nara Prefecture; Akashi City, Hyogo Prefecture; Hashimoto City, Wakayama Prefecture; and Iwata City, Shizuoka Prefecture) from September 2019 to August 2021. The following inclusion criteria were applied:

1. Each patient and his/her family agreed to participate in the study at the time of admission;
2. Each patient was aged ≥65 years;
3. An occupational therapist had determined the patient as being capable of understanding the questions.

Patients were excluded if they were considered to have difficulties in understanding due to severe cognitive impairment (an MMSE score of less than 10), had a decreased level of consciousness or mental function, or if the patient’s physician determined that the patient was at risk in participating in the survey [39]. This study also included oldest-old and cognitively impaired patients. Therefore, even when their cognitive abilities were considered adequate, questions were asked slowly in the quiet environment of the hospital room to ensure they were accurately comprehended by patients, according to their level of understanding.

### 3.3. Analysis

SPSS Version 26.0 was used for statistical analysis, and significance was set at <5% through a two-tailed test. First, the normality of the measurements was assessed using the Kolmogorov–Smirnov test. We confirmed that the distribution of scores for each rating scale did not follow a normal distribution. In addition, distortions in the distribution of item scores were checked. Items with the mean ± standard deviation (SD) exceeding the range and whose percentage of participants with maximum and minimum scores exceeded 15% were deleted using a previously reported method for assessing ceiling and floor effects [40,41].

Next, in the exploratory factor analysis for structural validity, we conducted a varimax rotation using the least squares method. In so doing, an eigenvalue >1 and a loading factor >0.4 were considered [42]. After these were eliminated, factor analysis was conducted again. At the time of factor determination, correlations between factors were tested using Spearman’s rank correlation coefficient. Confirmatory factor analysis was performed using the maximum likelihood method. Specifically, the goodness-of-fit of the models was compared in terms of the comparative fit index (CFI), goodness of fit index (GFI), adjusted goodness of fit index (AGFI), and root mean square error of approximation (RMSEA). The thresholds for these indices of goodness-of-fit were CFI > 0.90 and RMSEA < 0.08. In comparison, the thresholds for marginal fit were CFI > 0.87 and RMSEA values were <0.10 [43]. In assessing criteria-related validity, the correlation between the total score and each factor score of the PES-J and the total scores of the HLC, SE, SOC-13, RSES, and PGC was examined using Spearman’s rank correlation coefficient.

Reliability was assessed for internal consistency, test–retest reliability, and measurement error. For internal consistency, Cronbach’s alphas were calculated for the entire scale and for each factor group extracted in the exploratory factor analysis. For test–retest reliability, Spearman’s rank correlation coefficient was calculated for the total score, and the weighted kappa coefficient was calculated for each item in the first and second survey results. Furthermore, in the verification of measurement error, we calculated the standard error of measurement (SEM) and minimal detectable change (MDC) using the first SD and Spearman’s rank correlation coefficient between the first and second surveys [44,45].

### 3.4. Ethics

This study was approved by the appropriate university research ethics review committees and was conducted in accordance with the Declaration of Helsinki. The participants were given verbal and written explanations concerning participation, including their freedom to withdraw from the study at any time, that there would be no disadvantages incurred such as withdrawal of medical treatment, and that complete protection of personal information would be ensured. Subsequently, written informed consent was obtained.

## 4. Results

### 4.1. Participant Characteristics

A total of 153 patients consented to participate in the first survey. Of these, 151 completed both surveys (two dropped out as they were transferred back to hospital due to deteriorating conditions). The characteristics of the patients are listed in Table 1. Of these, 54 were male (35.76%) and the average age was 81.75 ± 7.15 years. In terms of illness, 33 patients were hospitalized for central nervous system diseases, 79 for orthopedic diseases, and 39 for disuse syndrome associated with medical diseases. Among the participants, 15 had diabetes mellitus (9.93%), 44 had hypertension (29.14%), and 45 had dementia (29.80%).

### 4.2. Verification of Ceiling and Floor Effects Using the Four-Point Scale

The results of the first survey are shown in Table 2. Questions with a ceiling effect included question 13, “Do staff force you to eat and drink even when you do not feel like doing so?” (−0.78 ± 0.84, 68 patients; 45.03% of the ceiling score) and question 40, “Do staff speak to you as if you are a child?” (−0.63 ± 0.71, 73 patients; 48.34% of the ceiling score). We re-examined the questions and deleted question 13, as its intent was already included in questions 12 and 28 (eating issues), although there are situations where such questions are necessary from the perspective of medical care for older patients. Question 40 was also deleted based on the results, as ethical concerns were expressed during the Japanese translation process. Thus, after verifying the ceiling and floor effects of the 40 items, 38 questions were included in the survey.

### 4.3. Structural Validity

Next, we conducted an exploratory factor analysis on the 38 items. The factor loading for question 25, “Do staff prevent you from making a decision on a treatment plan?” was 0.38. From the perspective of care, questions 19, “Do staff respect your decisions?” and 39, “Do staff make sure that you have made a clear decision?” appeared more appropriate for supporting the participant’s self-selection and self-determination. Therefore, question 25 was deleted. As a result, the survey retained 37 items (37-item PES-J) (−51 to 60 points), including 20 empowerment questions and 17 disempowerment questions.

In the exploratory factor analysis, six factors were extracted, with a cumulative contribution ratio of 66.14% (see Table 3). Factor 1 was labeled “Subject–staff interaction” comprising questions 2, 6, 16–18, 29, 32, 34–35, and 37, to capture staff communication and trust in terms of staff explanations and consent. Factor 2 was labeled “Environmental adjustment through collaboration” and comprised questions 3, 5, 8, 10, 15, 20, and 27, to capture the human and physical environment surrounding the person. Factor 3 was labeled “Necessary information gathering and problem awareness,” comprising questions 4, 7, 23, 26, 30, and 38, to capture any disclosure of information about the person’s illness and physical and mental functions. Factor 4 was labeled “Proactive behavioral practices based on self-selection and self-determination,” comprising questions 1, 9, 12, 28, and 31, to capture consideration of the person’s self-selection and self-determination in the execution and progress of activities. Factor 5 was labeled “Self-disclosure,” comprising questions 19, 21, 33, 36, and 39, to reflect the person’s opportunity for self-reflection and self-expression. Finally, factor 6 was labeled “Self-management of activities,” comprising questions 11, 14, 22, and 24, to capture the safety and privacy of the person. The Kaiser–Meyer–Olkin test result was 0.83, and Bartlett’s test of sphericity was significant at the 0.10% level, indicating a statistically valid structure. Furthermore, examination of correlations between factors did not reveal any strong correlations (see Table 4). In the results of confirmatory factor analysis, Faulkner’s two-factor model was statistically significant for the model (χ^2^ = 3244.53, df = 610, *p* < 0.01). Furthermore, the two-factor model clearly showed poor fit (CFI = 0.39, GFI = 0.41, AGFI = 0.32, RMSEA = 0.17). By contrast, the six-factor model constructed in this study was also statistically significant for the model (χ^2^ = 1251.62, df = 589, *p* < 0.01), but it may not be a reliable measure of model fit in this case. However, all factor loadings were significant at the 0.01 level. This six-factor model exhibited acceptable and validated model fit (CFI = 0.89, GFI = 0.81, AGFI = 0.75, and RMSEA = 0.08).

### 4.4. Criteria-Related Validity

All of the scales examined were significantly correlated with the PES-J total score and its six factors. The HLC score was 35.08 ± 4.29 (ρ = 0.41–0.79, *p* < 0.01); the SE score was 69.97 ± 13.13 (ρ = 0.43–0.78, *p* < 0.01); the SOC-13 score was 52.70 ± 7.54 (ρ = 0.23–0.46, *p* < 0.01); the RSES score was 33.60 ± 6.40 (ρ = 0.36–0.70, *p* < 0.01); and the PGC score was 9.20 ± 3.14 (ρ = 0.35–0.66, *p* < 0.01) (see Table 5).

### 4.5. Verification of Reliability

#### 4.5.1. Internal Consistency

Cronbach’s alpha was 0.93 for all 37 items. The Cronbach’s alphas for the six factors were 0.93, 0.91, 0.91, 0.92, 0.91, and 0.75, respectively.

#### 4.5.2. Test–Retest Reliability

In our test–retest of reliability, Spearman’s rank correlation coefficient between the first and second total scores was ρ = 0.96, *p* < 0.01. The weighted Kappa coefficients for the questions ranged between 0.52 and 0.90 (*p* < 0.01) (see Table 6).

#### 4.5.3. Measurement Error

The SD of the first survey was 15.97, and the intraclass correlation coefficient of the total score of the first and second surveys was 0.95, resulting in an SEM of 3.30. Furthermore, the MDC was 9.20.

## 5. Discussion

In this study, we translated the PES into Japanese and verified the reliability and validity of the Japanese version. The results show that the PES-J had sufficient validity and reliability as a measure of empowerment for older patients admitted to general hospitals, with 37 items and six factors finally determined. We discuss below in more detail the results of the study, along with the process of creating the Japanese version and validating its validity and reliability.

### 5.1. Development of the PES-J

We followed the procedures of the ISPOR task force to translate the PES for application in Japan. We found no confusion due to culture-specific expressions or proper nouns in translating the original PES. Additionally, we were able to confirm that the scale could be deployed effectively among older patients in Japan. The questions were also reviewed by a multidisciplinary team to determine whether they were appropriate for clinical practice in Japan, and any modifications were discussed with the author of the original PES. Ultimately, the PES-J was considered a faithful translation of the original version of the rating scale.

In the first survey, the questioning time and patient fatigue were low, indicating its applicability to older patients and that the scale could also be used by various healthcare professionals. Regarding the response measurement, Faulkner [17], the original author, indicated that a three-point scale would not accurately reflect a patient’s response and recommended the application of a four-point scale. In other empowerment scales, four- and five-point scales have been used [15,18]. In Japan, a four-point scale is recommended, as a five-point scale is difficult to use, with older adults reported to have a tendency to concentrate their responses on the midpoint (e.g., giving “Neither” as a response) [46].

### 5.2. Validity and Reliability of the 37-Item PES-J

As stated, an exploratory factor analysis applied to assess structural validity extracted six factors: subject–staff interaction, environmental adjustment through collaboration, necessary information gathering and problem awareness, proactive behavioral practices based on self-selection and self-determination, self-disclosure, and self-management of activities. Zimmerman [13] stated that empowerment comprises several components: (1) an internal individual component (an individual’s thoughts and beliefs), (2) an interactional component (interaction with the environment surrounding the individual), and (3) a behavioral component (actions that involve the community group or organization and build functional relationships). A recent study of older adults in Japan showed empowerment to be composed of six attributes [47]. Since empowerment as a concept involves diversity based on differences in culture and historical background, this may account for the difference in the number of factors in the 37-item PES-J and the original PES. Of the six factors identified in this study, factors 3 and 5, as internal components, capture awareness of the person’s current situation and challenges, while factors 1 and 2, as interactional components, capture interaction with others and the environment. Furthermore, factors 4 and 6 capture the practice and management of self-selected and self-determined behavior, the behavioral component. As such, this factor structure aligns with the components of empowerment proposed by Zimmerman [13]. In addition, Pekonen et al. [18] included “patient knowledge,” “patient coping ability,” “patient behavior,” and “support by others” as common concepts to be measured in a patient empowerment scale. In the 37-item PES-J, “patient knowledge” is captured in factors 3 and 5, “patient coping ability” in factors 3 and 6, “patient behavior” in factors 4 and 6, and “support by others” in factors 1 and 2. Based on the above, the scale can be considered a valid structure, as it encompasses the common concepts of empowerment presented by Zimmerman [13] and Pekonen et al. [18]. The results of confirmatory factor analysis also indicated that the six-factor structure obtained in this study was a more practical model than the two-factor structure in the original version. Contrarily, the model fit was not good in the six-factor structure either, remaining within the acceptable range. Based on these results, it was considered necessary to further revise the factor structure and question items, taking into account the complexity of the empowerment concept.

In the criteria validity assessment, the total score and scores for each factor of the 37-item PES-J were correlated with the HLC, SE, SOC-13, RSES, and PGC. Self-efficacy, self-esteem, locus of control, and sense of coherence have been clearly conceptualized and used as important means of measuring patient empowerment [11,24,25,48]. Furthermore, patient empowerment has been found to relate strongly to subjective satisfaction and improvement in quality of life [49]. Thus, our results indicated that the concept of empowerment, as measured in the 37-item PES-J, related well to the five concepts of locus of control, self-efficacy, sense of coherence, self-esteem, and subjective well-being as assessed in previous studies, thereby meeting the standards of external criteria.

Regarding internal consistency, Cronbach’s alphas, according to the exploratory factor analysis, were 0.93, 0.91, 0.92, 0.92, 0.91, and 0.75 for the six factors, respectively. Cronbach’s alpha is often lower as the number of items evaluated decreases, while seeking a criterion of 0.80 or higher [50]. Considering this, the 37-item PES-J exhibited high internal consistency. Furthermore, Cronbach’s alpha for all items in the 37-item PES-J was 0.93, indicating its unidimensionality as a scale to measure the empowerment of older patients.

In test–retest reliability assessment, the correlation between each total score of the first and second surveys was ρ = 0.96—a very high correlation. Furthermore, the weighted kappa coefficient for each item ranged from 0.52 to 0.90 (*p* < 0.01), ranging from moderate agreement to almost perfect or perfect agreement [51]. The original PES did not assess test–retest reliability; therefore, it is difficult to make comparisons. However, all items in this survey were above moderate agreement, so it can be considered sufficiently reliable as a measurement scale for older patients.

Finally, the measurement errors were 3.30 for the SEM and 9.20 for the MDC. The COSMIN checklist also indicates that it is desirable to clarify whether individual score changes are clinically meaningful changes [21,22]. In this study, it was determined in the 37-item PES-J that the criterion for a minimum clinically meaningful change (MCID) was 10 points, such that a change of >10 points for an individual could be interpreted as a meaningful change.

### 5.3. Application of the 37-Item PES-J to Health Promotion in Hospitalized Older Patients

By applying the 37-item PES-J to older inpatients, we were able to examine the factors that influence empowerment. Studies have shown that hospitalized older patients are at a high risk of developing learned helplessness due to decreased empowerment, leading to their becoming bedridden and confined [12,17]. Being bedridden and confined are important factors that inhibit functional recovery and lead to an increase in the need for greater family care [52,53]. However, by measuring the empowerment of hospitalized older patients, it is possible to clarify the effects of empowerment on physical activity and daily activities, which in turn can provide a basis for establishing better targeted care and support aimed at preventing inactivity and confinement.

In Japanese culture, older adults generally believe in following the wishes of their doctor or medical professional [5]. However, in recent years, shared decision-making and shared treatment goals among multiple medical professionals (e.g., nurses, physicians, therapists, and dietitians) and patients have become more important [54]. Labonté [55] showed that through interaction and acceptance, healthcare professionals can empower patient decisions and promote independence. Castro et al. [56] also showed that promoting empowerment among hospitalized older patients can have positive effects on the quality of care, increasing patient understanding and improving safety and satisfaction. Furthermore, Stichler and Pelletier [57] showed that empowerment facilitated patients’ readiness for discharge (activation).

In the future, the use of the 37-item PES-J is likely to facilitate medical professionals in engaging with and addressing environmental issues as perceived by patients. Thus, the 37-item PES-J can promote specific and better targeted life support and discharge preparation based on appropriately informed involvement with patient decision-making.

### 5.4. Limitations

This study examined the structural validity, criteria-related validity, cross-cultural validity, test–retest reliability, and standard error in the PES-J, which were not addressed in the original PES, to confirm its clinical usefulness. However, interrater reliability, responsiveness, and interpretability were not tested and should be tested in future research. Furthermore, this study included cognitive impaired and oldest-old patients, and it could not be said with certainty that the patients’ intentions and actions were reflected in the study. In addition, since the patients were in the early post-hospitalization period with illnesses, the effects of their relationship with the investigators and their physical condition, mood, and fatigue at the time of the study could not be completely confirmed or eliminated. These points require further validation by increasing the number of patients and including other factors that might affect individual empowerment. According to the COSMIN checklist, the target number of participants should be equal to the number of items × 10 [21,22]. Thus, the 37-item PES-J should be analyzed using a larger number of participants in future research.

## 6. Conclusions

In this study, we translated the PES into Japanese and assessed its validity and reliability. As a result, we were able to confirm that the 37-item PES-J is a valid and reliable scale and is, therefore, applicable to hospitalized older patients in Japan. Research on the empowerment of older patients in Japan is still in its infancy, with one reason being the underdevelopment of an evaluation scale. Thus, the use of this scale to measure the empowerment of older patients can contribute to the development of research on factors that influence their empowerment. This scale captures the care and environment experienced by older patients in terms of their subjective perspectives. It can be readily used not only by nurses but also by co-medical professionals such as occupational therapists and caregivers, which may lead to the sharing of care issues and goals by medical care teams and improvement in patient-centered care practices. Through using this tool, where the number of older adults is rapidly increasing, health professionals in various fields can contribute to creating a society where older adults can achieve a better quality of life by more effectively addressing the risks of confinement and inactivity.

## Figures and Tables

**Table 1:** Characteristics of participants.

Item		No./%	37-Item PES-J Score
Sex	Male	54 (35.76%)	27.20 (16.85)
	Female	97 (64.24%)	24.83 (15.56)
Age (years)		81.75 ± 7.15	25.60 (15.97)
Years of education		11.20 ± 1.81	25.60 (15.97)
Major physical diseases resulting in hospitalization (no. people)	Central nervous system disease	33 (21.85%)	25.39 (15.92)
	Orthopedic diseases	79 (52.32%)	26.52 (15.29)
	Disuse syndromes associated with medical diseases	39 (25.83%)	23.92 (17.57)
Presence of diabetes (no. people)	Affected	15 (9.93%)	30.07 (14.01)
	Unaffected	136 (90.07%)	25.11 (16.14)
Presence of hypertension (no. people)	Affected	44 (29.14%)	25.45 (17.69)
	Unaffected	107 (70.86%)	25.66 (15.30)
Presence of dementia (no. people)	Affected	45 (29.80%)	26.02 (14.52)
	Unaffected	106 (70.20%)	25.42 (16.61)

37-item PES-J: 37-item Patient Empowerment Scale—Japanese version.

**Table 2:** Measurement results of the PES-J.

Question	Content	Empowerment (E)/Disempowerment (D)	Min Score	Max Score	Mean	Std Dev.	Ceiling Effect (% of Maximum Value)	Floor Effect
1	Do staff make sure that you can reach a nurse?	E1	0	3	1.90	0.96	2.87	0.94
2	Do staff give you positive words that encourage you to achieve specific health goals?	E2	0	3	2.15	0.77	2.92	1.38
3	Do staff work quietly during the night so that you can sleep?	E3	0	3	2.11	0.81	2.92	1.31
4	Do staff provide you with information related to your conditions?	E4	0	3	1.92	0.88	2.80	1.05
5	Do staff move your bed and locker in your room against your preferences?	D1	−3	0	−0.81	0.76	−0.05	−1.57
6	Do staff refuse to address your concerns?	D2	−3	0	−0.90	0.79	−0.11	−1.69
7	Do staff clearly answer your care-related questions?	E5	0	3	1.93	0.88	2.81	1.05
8	Do staff respond promptly when you complain of pain?	E6	0	3	2.05	0.88	2.93	1.16
9	Do staff make noise and preventing you from sleeping at night?	D3	−3	0	−1.13	0.93	−0.19	−2.06
10	Do staff provide you with personal care assistance without obtaining permission from you?	D4	−3	0	−0.89	0.78	−0.12	−1.67
11	Do staff instruct you to participate in an activity against your wishes?	D5	−3	0	−0.81	0.67	−0.14	−1.48
12	Do staff take away food and drink from your table before you are finished eating and drinking?	D6	−3	0	−0.96	0.90	−0.06	−1.86
13	Do staff force you to eat and drink even when you do not feel like doing so?	D7	−3	0	−0.78	0.84	0.06 * (45.03%)	−1.62
14	Do staff violate your privacy while you are engaging in a personal activity?	D8	−3	0	−0.77	0.61	−0.16	−1.37
15	Do staff resolve your concerns?	E7	0	3	2.01	0.83	2.84	1.19
16	Are staff ready to help you at all times once they notice that you need help?	E8	0	3	2.13	0.80	2.93	1.32
17	Do staff appear to be busy with other work even when they notice that you need help?	D9	−3	0	−0.83	0.72	−0.12	−1.55
18	Do staff provide you with care without explaining to you about their actions?	D10	−3	0	−0.83	0.72	−0.11	−1.55
19	Do staff respect your decisions?	E9	0	3	2.06	0.81	2.87	1.25
20	Do staff mention personal information in places where their conversation can be heard by other patients?	D11	−3	0	−0.86	0.80	−0.06	−1.66
21	Do staff listen to what you need to say without interrupting you?	E10	0	3	2.13	0.83	2.96	1.30
22	Do staff make sure that you can carry out certain activities by yourself?	E11	0	3	2.05	0.83	2.88	1.22
23	Do staff check that you have understood information given to you?	E12	0	3	1.89	0.86	2.75	1.04
24	Do staff request you to carry out tasks which you cannot do due to your conditions?	D12	−3	0	−0.84	0.63	−0.21	−1.47
25	Do staff prevent you from making a decision on a treatment plan?	D13	−3	0	−0.85	0.73	−0.12	−1.59
26	Do staff give you information too fast for you to comprehend?	D14	−3	0	−1.04	0.74	−0.30	−1.78
27	Are staff slow in addressing your pain when you complain of it?	D15	−3	0	−0.87	0.81	−0.06	−1.67
28	Do staff give you sufficient time to finish eating and drinking before they take your food away?	E13	0	3	2.05	0.92	2.97	1.13
29	Do staff show you their understanding when you discuss your concerns with them?	E14	0	3	2.04	0.88	2.92	1.16
30	Do staff provide you with information related to options for your care going forward?	E15	0	3	1.82	0.88	2.70	0.94
31	Do staff understand the environment of your room?	E16	0	3	1.86	0.93	2.79	0.93
32	Do staff perform a treatment procedure without letting you know what it involves?	D16	−3	0	−0.79	0.71	−0.08	−1.50
33	Do staff give you sufficient time to answer questions?	E17	0	3	2.06	0.84	2.90	1.23
34	Are there any instances in which staff do not help you with tasks which you cannot carry out by yourself?	D17	−3	0	−0.86	0.70	−0.16	−1.56
35	Do staff say negative words which hurt your dignity (pride)?	D18	−2	0	−0.78	0.61	−0.16	−1.39
36	Do staff seek your permission before they start carrying out tasks?	E18	0	3	2.01	0.90	2.91	1.11
37	Do staff act arrogantly when they speak with you? (e.g., standing with hands on hips)	D19	−2	0	−0.71	0.64	−0.07	−1.35
38	Do staff always give you explanations about their actions involved in care operations?	E19	0	3	2.00	0.88	2.88	1.12
39	Do staff make sure that you have made a clear decision?	E20	0	3	2.11	0.81	2.92	1.30
40	Do staff speak to you as if you are a child?	D20	−3	0	−0.63	0.71	0.08 * (48.34%)	−1.34
Total score		-	−19	56	23.34	17.05	-	-

PES-J: Patient Empowerment Scale—Japanese version, * *p* < 0.05.

**Table 3:** Structural validity of the 37-item PES-J.

Factor	No.	Content	Factor 1	Factor 2	Factor 3	Factor 4	Factor 5	Factor 6	Communality
			Factor Loadings						
Factor 1 Subject–staff interaction	35	Do staff use negative words that hurt your dignity (pride)?	0.84	0.17	0.08	0.06	0.05	−0.06	0.89
	2	Do staff offer positive words which encourage you to achieve specific health goals?	0.77	0.05	0.20	0.21	0.01	0.09	0.92
	32	Do staff perform a treatment procedure without letting you know what it involves?	0.77	0.06	0.14	0.04	0.11	0.06	0.85
	18	Do staff provide you with care without explaining their actions?	0.76	0.05	0.14	0.03	0.18	0.15	0.80
	37	Do staff act arrogantly when they speak to you? (e.g., standing with hands on hips)	0.76	0.25	0.05	0.08	0.06	−0.07	0.78
	16	Are staff ready to help you at all times once they notice that you need help?	0.75	0.10	0.07	0.20	0.11	0.18	0.89
	34	Are there any instances in which staff do not help you with tasks which you cannot carry out by yourself?	0.75	0.10	−0.06	0.15	0.08	0.25	0.88
	6	Do staff refuse to address your concerns?	0.73	0.17	0.06	0.13	0.04	0.27	0.78
	17	Do staff appear to be busy with other work even when they notice that you need help?	0.70	0.04	0.06	0.16	0.18	0.12	0.81
	29	Do staff show you their understanding when you discuss your concerns with them?	0.66	−0.07	0.15	0.14	0.10	0.02	0.84
Factor 2 Environmental adjustment through collaboration	3	Do staff work quietly during the night so that you can sleep?	0.14	0.89	<−0.01	0.05	0.10	−0.05	0.92
	5	Do staff move your bed and locker in your room against your wishes?	0.08	0.85	0.04	0.05	0.09	0.15	0.90
	20	Do staff mention personal information in places where their conversation can be heard by other patients?	0.09	0.83	0.08	0.03	0.03	0.11	0.88
	15	Do staff resolve your concerns?	0.15	0.79	0.17	0.05	0.09	0.06	0.98
	27	Are staff slow in addressing your pain when you complain of it?	0.14	0.71	0.08	0.16	0.07	0.09	0.80
	10	Do staff provide you with personal care assistance without obtaining permission from you?	0.10	0.71	0.11	−0.06	0.05	0.22	0.78
	8	Do staff promptly respond when you complain of pain?	0.10	0.60	0.19	0.07	0.13	−0.05	0.76
Factor 3 Necessary information gathering and problem awareness	38	Do staff always give you explanations about their actions involved in care operations?	0.13	0.14	0.84	0.12	0.15	0.09	0.89
	7	Do staff clearly answer your care-related questions?	0.11	0.08	0.84	0.09	0.20	−0.13	0.92
	4	Do staff provide you with information related to your conditions?	0.08	0.13	0.83	0.14	0.17	−0.02	0.90
	23	Do staff check if you have understood information given to you?	0.12	0.04	0.82	0.04	0.26	0.02	0.87
	30	Do staff provide you with information related to options for your care going forward?	0.09	0.01	0.81	0.04	0.26	0.02	0.93
	26	Do staff give you information too fast for you to comprehend?	0.13	0.15	0.54	0.11	0.08	0.14	0.77
Factor 4 Proactive behavioral practices based on self-selection and self-determination	1	Do staff make sure that you can reach a nurse?	0.13	0.03	0.07	0.93	0.17	−0.03	0.94
	28	Do staff give you sufficient time to finish eating and drinking before they take it away?	0.11	0.03	0.05	0.90	0.10	−0.01	0.89
	31	Do staff understand the environment of your room?	0.15	<0.01	0.13	0.80	0.16	0.13	0.83
	9	Do staff make noise and prevent you from sleeping at night?	0.15	0.16	0.06	0.79	0.06	−0.03	0.82
	12	Do staff take away food and drink before you finish?	0.14	0.03	0.16	0.67	0.23	0.03	0.78
Factor 5 Self-disclosure	21	Do staff listen to what you need to say without interrupting you?	0.18	0.11	0.14	0.14	0.87	0.12	0.93
	19	Do staff respect your decisions?	0.16	0.02	0.21	0.08	0.81	0.15	0.83
	39	Do staff make sure that you have made a clear decision?	0.09	0.12	0.28	0.16	0.80	−0.06	0.84
	33	Do staff give you sufficient time to answer questions?	0.08	0.03	0.16	0.19	0.78	0.01	0.85
	36	Do staff seek your permission before they start carrying out tasks?	0.03	0.20	0.14	0.19	0.73	−0.13	0.86
Factor 6 Self-management of activities	11	Do staff instruct you to participate in an activity against your preference?	0.21	0.26	0.09	−0.07	−0.02	0.65	0.81
	22	Do staff make sure if you can carry out certain activities by yourself?	0.25	0.05	0.15	0.17	0.11	0.63	0.76
	14	Do staff violate your privacy while you are engaging in a personal activity?	0.22	0.22	0.09	−0.02	0.03	0.59	0.73
	24	Do staff request that you carry out tasks you cannot do due to conditions?	0.25	0.21	−0.04	−0.01	0.11	0.46	0.66
		Eigenvalue	10.65	4.23	3.96	2.99	2.18	1.72	
		Contributing ratio	16.74	12.61	11.42	10.33	9.90	5.14	
		Cumulative contribution ratio	16.74	29.35	40.77	51.10	61.00	66.14	

37-item PES-J: 37-item Patient Empowerment Scale—Japanese version.

**Table 4:** Correlation between factors of the 37-item PES-J.

	Correlation Coefficient					
	Factor 1	Factor 2	Factor 3	Factor 4	Factor 5	Factor 6
Factor 1	1	0.27 **	0.24 **	0.34 **	0.23 **	0.39 **
Factor 2	―	1	0.19 *	0.17 *	0.20 *	0.28 **
Factor 3	―	―	1	0.20 *	0.43 **	0.22 **
Factor 4	―	―	―	1	0.34 **	0.06
Factor 5	―	―	―	―	1	0.15
Factor 6	―	―	―	―	―	1

* *p* < 0.05, ** *p* < 0.01.

**Table 5:** Criteria-related validity of the 37-item PES-J.

Scale	Score (Standard Deviation)	Correlation Coefficient with PES-J						
		Total	Factor 1	Factor 2	Factor 3	Factor 4	Factor 5	Factor 6
HLC	39.29 (4.81)	0.79 **	0.62 **	0.49 **	0.46 **	0.47 **	0.41 **	0.41 **
SE	69.97 (13.13)	0.78 **	0.56 **	0.50 **	0.45 **	0.43 **	0.45 **	0.45 **
SOC-13	52.70 (7.55)	0.46 **	0.40 **	0.32 **	0.29 **	0.23 **	0.25 **	0.32 **
RSES	33.60 (6.41)	0.70 **	0.53 **	0.41 **	0.39 **	0.36 **	0.43 **	0.44 **
PGC	9.20 (3.15)	0.66 **	0.46 **	0.47 **	0.36 **	0.37 **	0.40 **	0.35 **

37-item PES-J: 37-item Patient Empowerment Scale—Japanese version; HLC: Health Locus of Control Scale; SE: Generalized Self-Efficacy Scale; SOC-13: 13-item Sense of Coherence Scale; RSES: Rosenberg Self Esteem Scale; PGC: Philadelphia Geriatric Center Morale Scale; ** *p* < 0.01.

**Table 6:** Test–retest reliability of the 37-item PES-J.

No.	(ICC_2,1_) Weighted Kappa Coefficient	Spearman’s Rank Correlation Coefficient	*p* Value	No.	(ICC_2,1_) Weighted Kappa Coefficient	Spearman’s Rank Correlation Coefficient	*p* Value
Total	-	0.96	<0.01	Question 20	0.72	-	<0.01
Question 1	0.78	-	<0.01	Question 21	0.64	-	<0.01
Question 2	0.70	-	<0.01	Question 22	0.70	-	<0.01
Question 3	0.81	-	<0.01	Question 23	0.77	-	<0.01
Question 4	0.90	-	<0.01	Question 24	0.81	-	<0.01
Question 5	0.82	-	<0.01	Question 26	0.74	-	<0.01
Question 6	0.71	-	<0.01	Question 27	0.80	-	<0.01
Question 7	0.65	-	<0.01	Question 28	0.82	-	<0.01
Question 8	0.61	-	<0.01	Question 29	0.80	-	<0.01
Question 9	0.78	-	<0.01	Question 30	0.65	-	<0.01
Question 10	0.71	-	<0.01	Question 31	0.81	-	<0.01
Question 11	0.52	-	<0.01	Question 32	0.73	-	<0.01
Question 12	0.84	-	<0.01	Question 33	0.72	-	<0.01
Question 14	0.69	-	<0.01	Question 34	0.53	-	<0.01
Question 15	0.66	-	<0.01	Question 35	0.82	-	<0.01
Question 16	0.64	-	<0.01	Question 36	0.61	-	<0.01
Question 17	0.72	-	<0.01	Question 37	0.76	-	<0.01
Question 18	0.60	-	<0.01	Question 38	0.64	-	<0.01
Question 19	0.65	-	<0.01	Question 39	0.65	-	<0.01

37-item PES-J: 37-item Patient Empowerment Scale—Japanese version.

## Data Availability

Due to privacy and ethical concerns, neither the data nor the data source can be made available.
